# Supplementary material for: Uncovering a ‘sensitive window’ of multisensory and motor neuroplasticity in the cerebrum and cerebellum of male and female starlings
Source: eLife. 2021 Jun 7;10:e66777. doi: 10.7554/eLife.66777 (PMC8219385; doi:10.7554/eLife.66777)
Supplement: Transparent reporting form [file elife-66777-transrepform.pdf]

eLIFE
1st Floor
24 Hills Road
Cambridge CB2 1JP, UK
P 01223 855340
W elifesciences.org
T @elife

eLife's transparent reporting form

We encourage authors to provide detailed information *within their submission* to facilitate the interpretation and replication of experiments. Authors can upload supporting documentation to indicate the use of appropriate reporting guidelines for health-related research (see EQUATOR Network), life science research (see the BioSharing Information Resource), or the ARRIVE guidelines for reporting work involving animal research. Where applicable, authors should refer to any relevant reporting standards documents in this form.

If you have any questions, please consult our Journal Policies and/or contact us: editorial@elifesciences.org.

Sample-size estimation

- You should state whether an appropriate sample size was computed when the study was being designed
- You should state the statistical method of sample size computation and any required assumptions
- If no explicit power analysis was used, you should describe how you decided what sample (replicate) size (number) to use

Please outline where this information can be found within the submission (e.g., sections or figure legends), or explain why this information doesn't apply to your submission:

A prior DTI study performed at our lab (De Groof, Verhoye et al. 2008, Orije, Cardon et al. 2020) included 9 starlings to statistically detect seasonal differences. As we propose a longitudinal study design with 6 time points, we have to take into account that animals might drop out due to several reasons e.g. birds might die during the experiment or birds might not respond well to anesthesia and consequently cannot be scanned. We foresee a failure rate of 10%, which corresponds to 1-2 subjects. For the neural correlate analysis, we used repeated measures correlation (rmcorr), which generally has higher power than a simple regression with averaged data (Bakdash and Marusich 2017). The number of animals used in this experiment was approved by the ethical Committee on Animal Care and Use of the University of Antwerp, Belgium.

Replicates

- You should report how often each experiment was performed
- You should include a definition of biological versus technical replication
- The data obtained should be provided and sufficient information should be provided to indicate the number of independent biological and/or technical replicates
- If you encountered any outliers, you should describe how these were handled
- Criteria for exclusion/inclusion of data should be clearly stated
- High-throughput sequence data should be uploaded before submission, with a private link for reviewers provided (these are available from both GEO and ArrayExpress)

Please outline where this information can be found within the submission (e.g., sections or figure legends), or explain why this information doesn't apply to your submission:

All information can be found in the Materials and Methods section.

Statistical reporting

- Statistical analysis methods should be described and justified
- Raw data should be presented in figures whenever informative to do so (typically when N per group is less than 10)
- For each experiment, you should identify the statistical tests used, exact values of N, definitions of center, methods of multiple test correction, and dispersion and precision measures (e.g., mean, median, SD, SEM, confidence intervals; and, for the major substantive results, a measure of effect size (e.g., Pearson's r, Cohen's d)
- Report exact p-values wherever possible alongside the summary statistics and 95% confidence intervals. These should be reported for all key questions and not only when the p-value is less than 0.05.

Please outline where this information can be found within the submission (e.g., sections or figure legends), or explain why this information doesn't apply to your submission:

Information regarding the statistical analyses can be found in the Materials and Methods section. Voxel-based statistical analyses are accompanied by respective table reporting on the cluster and peak level significance (figure 3, table 2; figure 5, table 3; figure 7, table 4; figure 11, table 5). Other statistical results are reported in the Results section following APA guidelines.

(For large datasets, or papers with a very large number of statistical tests, you may upload a single table file with tests, Ns, etc., with reference to sections in the manuscript.)

Group allocation

- Indicate how samples were allocated into experimental groups (in the case of clinical studies, please specify allocation to treatment method); if randomization was used, please also state if restricted randomization was applied
- Indicate if masking was used during group allocation, data collection and/or data analysis

Please outline where this information can be found within the submission (e.g., sections or figure legends), or explain why this information doesn't apply to your submission:

Experimental groups were determined based on sex and brain size. The latter is further explained in the Results section 3.3 and the Materials and Methods section 5.6.2.

Additional data files ("source data")

- We encourage you to upload relevant additional data files, such as numerical data that are represented as a graph in a figure, or as a summary table
- Where provided, these should be in the most useful format, and they can be uploaded as "Source data" files linked to a main figure or table
- Include model definition files including the full list of parameters used
- Include code used for data analysis (e.g., R, MatLab)
- Avoid stating that data files are "available upon request"

Please indicate the figures or tables for which source data files have been provided:

Source data is provided for figure 3-6, 8-11.

eLife Sciences Publications, Ltd is a limited liability non-profit non-stock corporation incorporated in the State of Delaware, USA, with company number 5030732, and is registered in the UK with company number FC030576 and branch number BR015634 at the address 1st Floor, 24 Hills Road, Cambridge CB2 1JP | August 2014

Bakdash, J. Z. and L. R. Marusich (2017). "Repeated Measures Correlation." Front Psychol **8**: 456.
De Groof, G., M. Verhoye, V. Van Meir, J. Balthazart and A. Van der Linden (2008). "Seasonal rewiring of the songbird brain: an in vivo MRI study." Eur J Neurosci **28**(12): 2475-2485; discussion 2474.
Orije, J., E. Cardon, G. de Groof, J. Hamaide, E. Jonckers, S. Van Massenhoven, V. Darras, J. Balthazart, M. Verhoye and A. Van der Linden (2020). "In vivo online monitoring of testosterone-induced neuroplasticity in a female songbird." Horm Behav **118**.

eLife Sciences Publications, Ltd is a limited liability non-profit non-stock corporation incorporated in the State of Delaware, USA, with company number 5030732, and is registered in the UK with company number FC030576 and branch number BR015634 at the address 1st Floor, 24 Hills Road, Cambridge CB2 1JP | August 2014
